# A Pilot Study of Microbial Succession in Human Rib Skeletal Remains during Terrestrial Decomposition

**DOI:** 10.1128/mSphere.00455-21

**Published:** 2021-07-14

**Authors:** Heather Deel, Alexandra L. Emmons, Jennifer Kiely, Franklin E. Damann, David O. Carter, Aaron Lynne, Rob Knight, Zhenjiang Zech Xu, Sibyl Bucheli, Jessica L. Metcalf

**Affiliations:** a Program in Cell & Molecular Biology, Colorado State University, Fort Collins, Colorado, USA; b Department of Animal Sciences, Colorado State University, Fort Collins, Colorado, USA; c Department of Biological Sciences, Sam Houston State University, Huntsville, Texas, USA; d Defense POW/MIA Accounting Agency Laboratory, Offutt AFB, Nebraska, USA; e Laboratory of Forensic Taphonomy, Forensic Sciences Unit, Chaminade University of Honolulu, Honolulu, Hawaii, USA; f School of Natural Sciences and Mathematics, Chaminade University of Honolulu, Honolulu, Hawaii, USA; g Center for Microbiome Innovation, University of California San Diego, La Jolla, California, USA; h Department of Pediatrics, University of California San Diego, La Jolla, California, USA; i Department of Computer Science and Engineering, University of California San Diego, La Jolla, California, USA; j Department of Bioengineering, University of California San Diego, La Jolla, California, USA; University of Wisconsin-Madison

**Keywords:** vertebrate decomposition, forensics, succession, microbiome, bone, taphonomy

## Abstract

The bones of decomposing vertebrates are colonized by a succession of diverse microbial communities. If this succession is similar across individuals, microbes may provide clues about the postmortem interval (PMI) during forensic investigations in which human skeletal remains are discovered. Here, we characterize the human bone microbial decomposer community to determine whether microbial succession is a marker for PMI. Six human donor subjects were placed outdoors to decompose on the soil surface at the Southeast Texas Applied Forensic Science facility. To also assess the effect of seasons, three decedents were placed each in the spring and summer. Once ribs were exposed through natural decomposition, a rib was collected from each body for eight time points at 3 weeks apart. We discovered a core bone decomposer microbiome dominated by taxa in the phylum *Proteobacteria* and evidence that these bone-invading microbes are likely sourced from the surrounding decomposition environment, including skin of the cadaver and soils. Additionally, we found significant overall differences in bone microbial community composition between seasons. Finally, we used the microbial community data to develop random forest models that predict PMI with an accuracy of approximately ±34 days over a 1- to 9-month time frame of decomposition. Typically, anthropologists provide PMI estimates based on qualitative information, giving PMI errors ranging from several months to years. Previous work has focused on only the characterization of the bone microbiome decomposer community, and this is the first known data-driven, quantitative PMI estimate of terrestrially decomposed human skeletal remains using microbial abundance information.

**IMPORTANCE** Microbes are known to facilitate vertebrate decomposition, and they can do so in a repeatable, predictable manner. The succession of microbes in the skin and associated soil can be used to predict time since death during the first few weeks of decomposition. However, when remains are discovered after months or years, often the only evidence are skeletal remains. To determine if microbial succession in bone would be useful for estimating time since death after several months, human subjects were placed to decompose in the spring and summer seasons. Ribs were collected after 1 to 9 months of decomposition, and the bone microbial communities were characterized. Analysis revealed a core bone decomposer microbial community with some differences in microbial assembly occurring between seasons. These data provided time since death estimates of approximately ±34 days over 9 months. This may provide forensic investigators with a tool for estimating time since death of skeletal remains, for which there are few current methods.

## INTRODUCTION

Terrestrial microbial decomposition of vertebrate remains includes a succession of communities of microbes from across the tree of life. Recent research has revealed that this succession can be repeatable and predictable enough that the composition of microbes can be used for estimating time since death, or postmortem interval (PMI) (1–6), which could be a useful tool for medicolegal investigations. Most microbial decomposition research has focused on time frames immediately following death using sample types such as the skin or other organs of the decedent and/or the associated soil. However, at later time frames of decomposition, often the only sample types available from the decedent are bones and teeth. Research in this area has revealed a potential use of microbial succession in bone for predicting PMI (7, 8), but more information is needed about the accuracy of PMI estimates for determining whether this could be a useful tool in medicolegal investigations. Furthermore, a more in-depth study of the bone decomposer bacterial and microbial eukaryotic communities would be useful for fields of anthropology, archaeology, paleontology, and ancient DNA.

Although decomposition is a continuous process, a decomposing body goes through visible changes that can be categorized into stages that are based on taphonomic landmarks (e.g., bloating) but are also related to changes in microbial processes. In the fresh stage, there are few visible changes to the decedent, with a cascade of biochemical events occurring at the cellular level (9). These events lead to discoloration, bloating, and the purging of fluids in the active decay stage, in which microbial processes are at their peak activity and decomposition is rapid. When the availability of nutrients for microbes decreases and the rate of decomposition declines, the decedent enters the advanced decay stage, in which most of the flesh is gone and there is some bone exposure. When at least 50% of the soft tissues are gone from the remains, the skeletonization stage is reached (10).

To characterize the human bone decomposer microbial community during skeletonization, we placed six human donor subjects to decompose at the Southeast Texas Applied Forensic Science Facility (three in the spring and three in the summer) in Huntsville, TX. Our goals were to understand the source of the microbial community and how it may differ across seasons and to determine whether microbial succession of decomposing bone could be used to estimate PMI. By placing decedents in two seasons, we sought to capture microbial succession within different environmental conditions, which could possibly affect the accuracy of PMI estimation models (11). Once naturally exposed (at least partially; see Materials and Methods), an entire rib was collected from each decedent at eight time points, and 16S rRNA and 18S rRNA amplicon sequencing from DNA extracted from a sectioned, pulverized piece of the rib was used to characterize succession of the bacterial and microbial eukaryotic communities. Bayesian source tracking was used to predict the source of the bone bacterial decomposer community, and a random forest regression was used for predicting PMI.

## RESULTS AND DISCUSSION

### Progression of decomposition and rib sampling.

In this study, we use Megyesi’s system of total body scoring (TBS) (10) based on the decomposition stages outlined in reference 12 to delineate between stages of decomposition. Photographs of the first 21 days of decomposition were used to calculate TBS and define fresh, active decay and advanced decay stages. Each of the decedents reached the advanced decay stage within 8 to 11 days after placement. Since photographs were not available after the first 21 days, we use the first known occurrence of rib exposure as the beginning of skeletonization. Rib exposure, and sample initiation, began within approximately 4 weeks for the spring placement subjects and within a range of approximately 4 to 6 weeks for the summer placement subjects (see Table S1). Sample initiation occurred within a range of 592 to 1,151 accumulated degree days (ADD), which is a temperature-based temporal scale (see Materials and Methods). Total sample collection ranged within the time frame of around 1 to 9 months of decomposition. Generally, we defined the stages of decomposition as fresh (0 to 6 TBS, ∼<50 ADD), active decay (6 to 17 TBS, ∼50 to 200 ADD), advanced decay (17 to 35 TBS, ∼200 to 600 ADD), and skeletonization (>35 TBS, ∼ >600 ADD). Decomposition stages often overlap and are not always clearly defined, and this experiment was no exception. However, using the TBS system and visual clues of skeletonization allowed us to place our sampling time frame of decomposition primarily within early skeletonization.

10.1128/mSphere.00455-21.8 TABLE S1 Summary of rib bones collected from human cadavers placed at STAFS. The beginning date of the range of collection indicates the first known occurrence of rib exposure. Download Table S1, DOCX file, 0.01 MB. Copyright © 2021 Deel et al. 2021 Deel et al. https://creativecommons.org/licenses/by/4.0/ This content is distributed under the terms of the Creative Commons Attribution 4.0 International license.

### Quality of amplicon sequence data.

In the 16S rRNA data set, a total of 1,270,545 reads were generated. After filtering out amplicon sequence variants (ASVs) with taxonomy assignments to mitochondria or chloroplast, there were 1,270,507 reads total with an average of 21,175 reads per sample, with sample sequence reads ranging from 712 to 39,169. We rarified the 16S rRNA data at 17,098 reads as an optimal balance for retaining both reads and samples, as this number was the count of the lowest-count sample necessary to include all but three samples. For the 18S rRNA data set, a total of 20,838,649 reads were generated. After filtering (see Materials and Methods), there were 18,178,587 reads total with an average of 288,549 reads per sample, with sample sequence reads ranging from 2,610 to 802,658. We rarified the 18S rRNA data at 214,940 sequences per sample, again, to optimize retaining both reads and samples in the data set, resulting in a loss of 10 samples. The taxonomic composition of negative controls can be seen in Fig. S1.

10.1128/mSphere.00455-21.1 FIG S1 (A and B) Taxonomic composition of negative controls of 16S rRNA (A) and 18S rRNA (B) datasets. Rare taxa include those with a mean relative abundance of 0.005 or lower in both plots. We included 15 negative extraction controls, of which 12 had enough detectable signal to be included in the sequencing pool of the 16S rRNA data. These 12 samples (A) averaged 387 reads, ranging from 4 to 2,232 reads. In the 18S rRNA data (B), there was an average of 12,187 reads per sample, ranging from 1,189 to 39,663 reads. Download FIG S1, TIF file, 2.8 MB. Copyright © 2021 Deel et al. 2021 Deel et al. https://creativecommons.org/licenses/by/4.0/ This content is distributed under the terms of the Creative Commons Attribution 4.0 International license.

### What microbes invade the bone, and where are they coming from?

**FIG 1:**
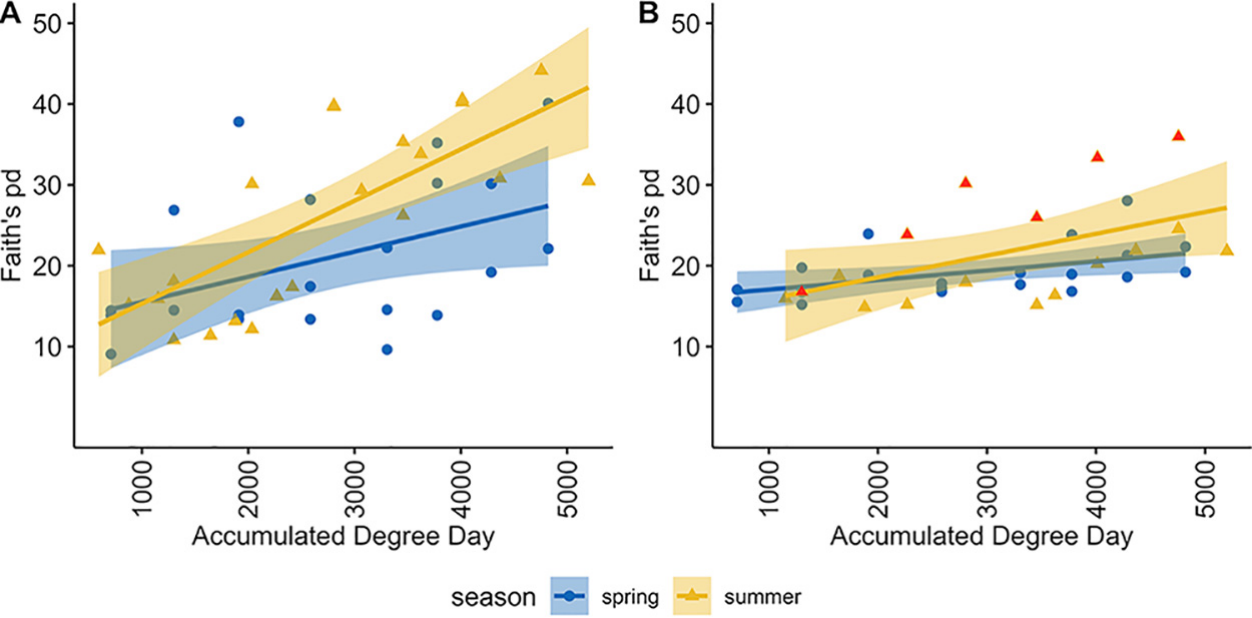
(A and B) A measure of alpha diversity using Faith’s phylogenetic diversity index with increasing ADD for 16S rRNA (A) and 18S rRNA (B) data sets. Red values are for visualization purposes and represent a single individual (064). Shaded areas around the line represent 95% confidence intervals. Linear mixed effects, *P* = 0.01 and *P* = 0.002 over ADD for 16S rRNA and 18S rRNA data sets, respectively.

We suspected that the diversity of microbes invading bone would increase as decomposition progressed, as more microbes from the environment (e.g., including the decedent and the surrounding soil) could likely access the internal bone as its structural integrity eroded. Our hypothesis was supported; the linear mixed effects model (which incorporates repeated measures) showed that there was a significant difference across time (ADD) for both bacteria and microbial eukaryotes (*P = *0.01 and *P = *0.002, respectively) (Fig. 1A and B). However, the positive trend in Faith’s phylogenetic diversity (PD) in the 18S rRNA data set appeared to be largely driven by a single individual (064), which is shown in red in Fig. 1B. We discovered two potential alpha diversity data point outliers via volatility plots and Q-Q normality plots, which were removed prior to linear mixed effects modeling of the 16S rRNA data (065.R11 and 011.L08). Additionally, a single potential outlier was removed from the 18S rRNA data (064.R12). Kruskal-Wallis effect size calculations between seasons, hosts, and the 1st and last ADDs showed that ADD had the highest effect on alpha diversity in all cases but one for both 16S rRNA and 18S rRNA data sets (Table S3).

10.1128/mSphere.00455-21.10 TABLE S3 Effect size calculations for alpha (Faith’s PD and Shannon index) and beta (weighted and unweighted UniFrac) diversity of the 16S rRNA and 18S rRNA data. The effect sizes (eta squared) of alpha diversity metrics were calculated using the H-value output of the Kruskal-Wallis test and the equation given by kruskal_effsize() in R. Effect sizes of beta diversity metrics are reported as pseudo-F values, which were provided in the beta-group-significance PERMANOVA outputs in QIIME2. Effect sizes were calculated for between seasons, hosts, and the first and last ADDs. Results are reported for calculations including both seasons as well as within season. Note that since effect size calculations were different between alpha and beta diversity metrics, results should only be compared within alpha or within beta diversity, but not between. Download Table S3, DOCX file, 0.02 MB. Copyright © 2021 Deel et al. 2021 Deel et al. https://creativecommons.org/licenses/by/4.0/ This content is distributed under the terms of the Creative Commons Attribution 4.0 International license.

**FIG 2:**
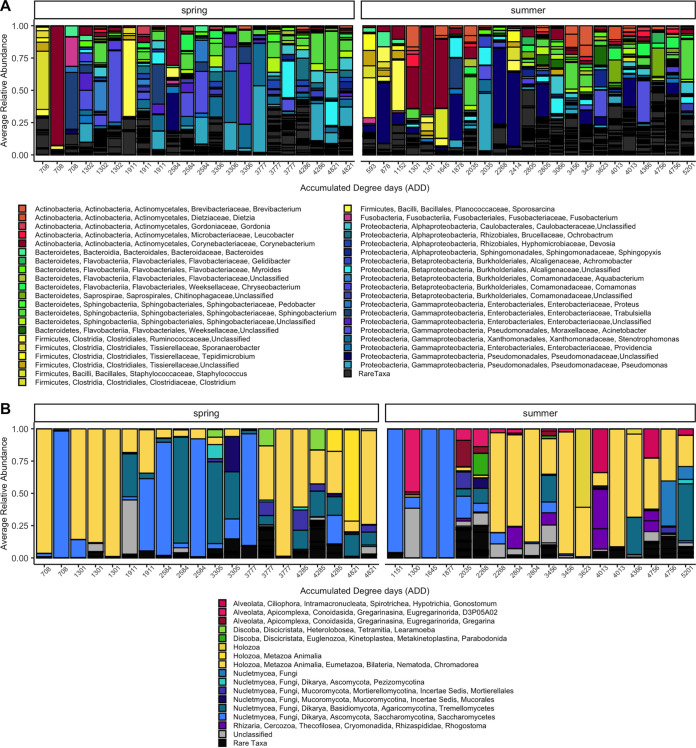
(A and B) Relative abundance taxa plots of the bacterial communities (A) and microbial eukaryotic communities (B). Rare taxa include those with a mean relative abundance of 0.005 or lower within the entire data set. Unclassified features shown in panel B generally include those that were only able to be classified as Eukaryota, with approximately 14% of all unclassified features identified as Opisthokonts.

Exploring the taxonomic composition of the bone microbial decomposer communities revealed many similar taxa that were widely represented across rib bone samples, regardless of ADD or season (Fig. 2). Core-feature analysis showed that the core bacterial phyla included *Proteobacteria*, *Firmicutes*, *Actinobacteria*, and *Bacteroidetes*. In the core microbiome, each phylum was represented by several features, with most of those features (22 out of 42 total core bacteria) being within the phylum *Proteobacteria*. The top five most prevalent core features (at the lowest identifiable level) included *Corynebacterium*, *Pseudomonadaceae*, Trabulsiella farmeri, Sphingobacterium mizutaii, and *Stenotrophomonas*. Outside of our defined core bacterial community, there were hundreds of different bacterial species, most of which had very low relative abundances. There were 86 total core microbial eukaryotic features. The top five most prevalent represented phyla (or subdivisions) included Ascomycota, Nematoda, Basidiomycota, Apicomplexa, and Ochrophyta. The top five most prevalent core features at the lowest identifiable level included two orders of Rhabditida, Debaryomycetaceae, Apiotrichum, and Candida bombi. Many of these core bacterial and microbial eukaryotic taxa have been discovered in other decomposer bone (7, 13), skin (1, 4, 14), and vertebrate decomposition-associated soil (1, 4, 15–17) microbial communities or have been found to decompose plant material and rotting wood (18, 19). There are likely many different processes occurring in this community, including the degradation and recycling of carcass-derived nutrients (20) and symbiotic relationships between core organisms. For example, some members of the community could degrade bone (21, 22), including Pseudomonas (23) and *Clostridium* organisms by releasing collagenases to break down bone collagen. Nematodes within the order Rhabditida likely display their saprophagous characteristics and feed off bacterial biomass and the decaying organic matter provided by the decedent (24, 25). Ochrophyta is an alga that has shown evidence of a symbiotic relationship with wood- and leaf litter-decomposing fungi such as Basidiomycota through acquiring carbon dioxide and protection from the sun while providing the fungi with a source of carbon and nitrogen (19). Additionally, some nonselected microbial processes known to occur in other environments, such as the soil, may also occur in the bone and contribute to the surrounding ecosystem. For example, decomposing bone taxa in the family *Pseudomonadaceae* likely contain phosphate-solubilizing bacteria that convert unavailable phosphorus into more accessible forms to be used by the surrounding soil and vegetation (26). Although this is not a comprehensive list of possible functional roles for these microbes within the bone decomposer community, it begins to represent the vast array of processes that require further investigation.

**FIG 3:**
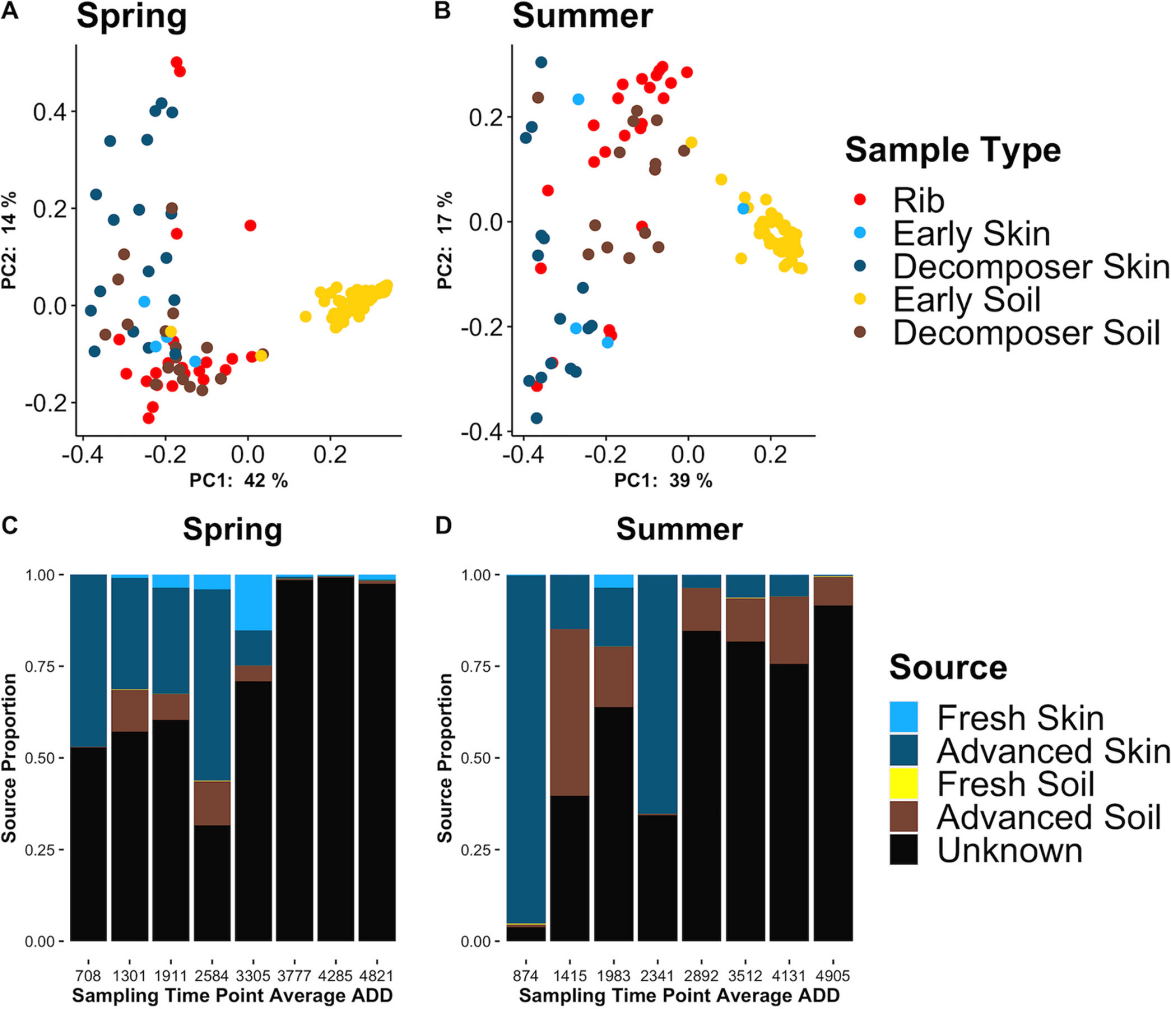
(A and B) Principal-coordinate analysis of 16S rRNA rib and source communities using the weighted UniFrac distance metric in the spring (A) and summer (B) placements. Spring pairwise PERMANOVA, q = 0.041 for rib and fresh skin comparison, and q = 0.001 for all other spring comparisons. Summer pairwise PERMANOVA, q = 0.002 for rib and fresh skin comparison, and q = 0.001 for all other summer comparisons. There were 999 permutations for all comparisons. (C and D) Succession of predicted portions of the fresh skin and soil (days 1 and 2) and advanced decay (days 19, 20, and 21) communities of the 16S rRNA spring (C) and summer (D) placements. Samples are grouped into collection time points 1 to 8.

To better understand the source of rib decomposer microbes, we compared the rib microbial decomposer communities (∼1 to 9 months after placement) to samples collected in earlier stages of decomposition for the same cadavers, including fresh (days 1 and 2 after placement) and advanced decay (days 19 to 21 after placement) skin and soil communities, as well as control soils that were not associated with a cadaver. The alpha diversity of decomposed rib bone bacterial communities was similar to those recovered from fresh skin and active decay skin and soil, which were all significantly lower than the alpha diversity for soils not associated with decomposing cadavers (Fig. S2A and B). The most prevalent taxa at the class level within the rib decomposer communities of both seasons were *Gammaproteobacteria* and *Actinobacteria*, with the most prevalent taxa comprising these classes being an unclassified *Pseudomonadaceae*, Pseudomonas, Acinetobacter, and two different *Corynebacterium* species. Of these common taxa, all were observed within the fresh and advanced decay cadaver skin and soil potential source communities (Fig. S3), with *Corynebacterium* species being found primarily in the summer placement communities. However, the composition of bone communities appeared distinct from the skin and soil source communities, particularly if abundance was considered (Fig. 3A and B, Fig. S2C and D).

10.1128/mSphere.00455-21.2 FIG S2 (A and B) 16S rRNA alpha diversity of the rib and source communities in the spring (A) and summer (B) placements, measured by Shannon index. (C and D) Principal-coordinate analysis of rib and potential source community 16S rRNA data using the unweighted UniFrac distance metric in the spring (C) and summer (D) placements. Download FIG S2, TIF file, 1.9 MB. Copyright © 2021 Deel et al. 2021 Deel et al. https://creativecommons.org/licenses/by/4.0/ This content is distributed under the terms of the Creative Commons Attribution 4.0 International license.

10.1128/mSphere.00455-21.3 FIG S3 (A and B) Taxa plots of the 16S rRNA spring (A) and summer (B) placement source data collected from days 1, 2, 19, 20, and 21 of decomposition (not including soil controls; see Fig. S6 for these data). Rare taxa include those with a mean relative abundance of 0.002 or lower in the spring and 0.003 or lower in the summer. Download FIG S3, TIF file, 2.8 MB. Copyright © 2021 Deel et al. 2021 Deel et al. https://creativecommons.org/licenses/by/4.0/ This content is distributed under the terms of the Creative Commons Attribution 4.0 International license.

Despite the unique composition of rib bone decomposer communities, Bayesian source tracking did predict a proportion of sources from the skin and soil advanced decay decomposer communities and a small proportion of the source from the fresh skin communities (Fig. 3C and D). These results suggest that rib bone decomposer communities are distinct from skin and soil communities (fresh or decomposition-associated), but likely originate, at least to some extent, from the surrounding environment of decomposing skin as well as soil bacteria. The rib bone environment likely selects a subset of microbes from cadaver skin and soil communities that are able to invade the bone and extract nutrients (27). More investigation is needed to determine whether additional microbial source(s) (e.g., nearby vegetation, rainfall, scavengers, insects) of the bone decomposer community exist.

### Is there a difference in the microbial community assembly in ribs from cadavers placed during different seasons?

**FIG 4:**
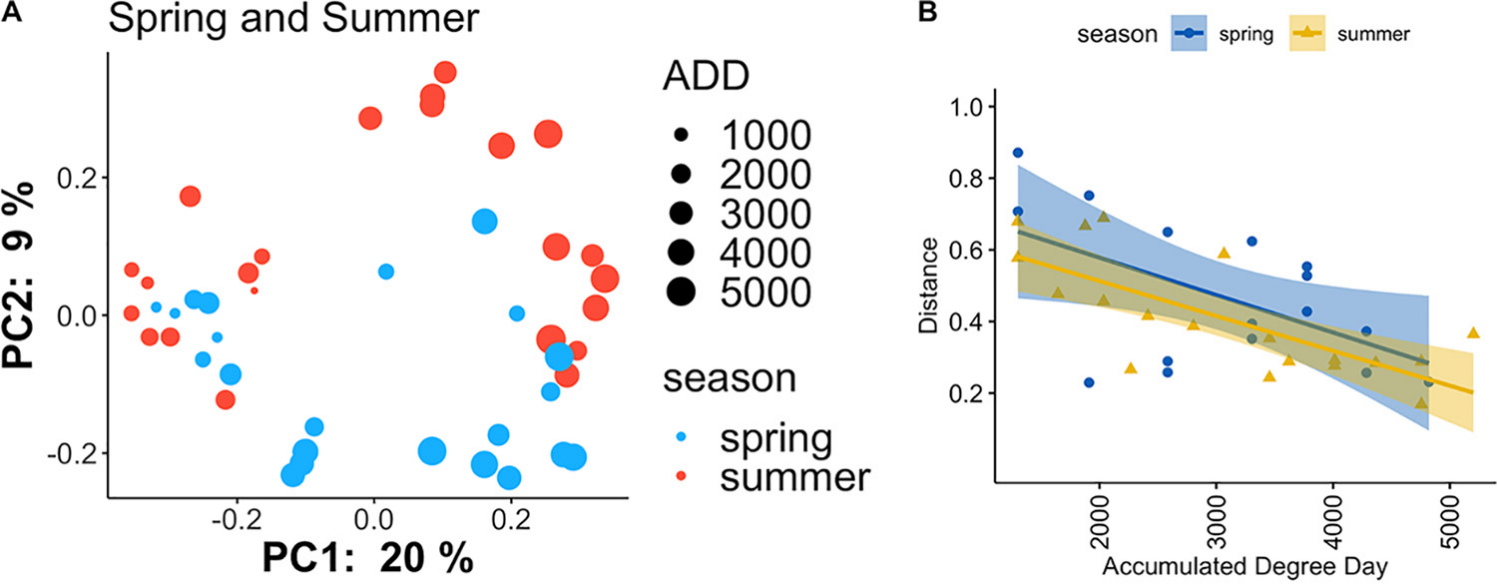
(A) A measure of beta diversity of 16S rRNA data using the unweighted UniFrac distance metric in both seasonal placements (PERMANOVA between seasons, *P = *0.004, pseudo-F [effect size] = 2.32, df = 1, with 999 permutations). (B) Scatterplot of linear mixed effects model input (ADD *P* = 0.012) in which distance is the rate of change of weighted UniFrac distances within season utilizing repeated measures within subjects. Shaded areas around the line represent 95% confidence intervals.

Although we discovered similar microbial taxa across all decomposed rib bones, regardless of seasonal placement (Fig. 2), there were significant differences in overall composition of bacterial and microbial eukaryotic communities in rib bones by season of placement (Fig. 4A, Fig. S4). We discovered differential abundance/presence of an unclassified *Pseudomonadaceae* at the genus level, Ochrobactrum intermedium at the species level, and an unclassified *Stenotrophomonas* at the ASV level, which were also confirmed as drivers of beta diversity patterns using robust Aitchison PCA (28) via EMPeror biplots (Fig. S5). In the microbial eukaryotic communities, differences between rib bone microbial communities for bodies placed during different seasons included *Learamoeba* and an unclassified Eumetazoa at level seven, and an unclassified Colpodea at level eight. These differences in assembly between seasons may be explained by multiple factors. Given that the soil decomposer community is a source of microbes within decomposed bone (Fig. 3C and D), we hypothesize that differences in soil composition between seasons may be a factor contributing to variation in microbial assembly within decomposed bone. We see evidence of this in our data when observing the beta diversity and taxonomic composition of soil control (not associated with decomposition) microbial communities collected within the first 21 days of decomposition (Fig. S6). This is unsurprising, as there is a wealth of evidence indicating that seasonality impacts microbial community composition (29–32). Variations in insect activity between seasons may also affect microbial assembly, as it is known that insects are less active in cooler seasons (9). Although ADDs were not very different between placements (Table S1), differences in temperature fluctuations over time between seasons may affect the relationship between insects and bacteria, fungi, protozoa, and nematodes (9), resulting in varied microbial community composition. Other differences in assembly between the spring and summer placements could be explained by variation in water content. While the amount of precipitation does not drastically differ between seasonal placements, the summer placement shows a higher accumulation of humidity (Fig. S7). This may contribute to an increase in water content and subsequently the microbial composition (30) of the summer placement soil that acts as a source of the bone microbes. General differences in environmental stability may also contribute to these differences. For example, there is greater diurnal temperature variation in spring and fall than in winter and summer, which would likely place constraints on bone microbial community assembly throughout our sampling time frame.

10.1128/mSphere.00455-21.4 FIG S4 (A) A measure of beta diversity of 18S rRNA data using the unweighted UniFrac distance metric (PERMANOVA between seasons *P* = 0.004, pseudo-F [effect size] = 3.07, df = 1 with 999 permutations). (B) A measure of beta diversity of 18S rRNA data using the weighted UniFrac distance metric. Shaded areas around the line represent 95% confidence intervals. Linear mixed effects across ADD *P* = 0.109. Download FIG S4, TIF file, 2.6 MB. Copyright © 2021 Deel et al. 2021 Deel et al. https://creativecommons.org/licenses/by/4.0/ This content is distributed under the terms of the Creative Commons Attribution 4.0 International license.

10.1128/mSphere.00455-21.5 FIG S5 Principal-coordinate analysis of the rib sample 16S rRNA data using the Aitchison distance metric from bodies placed in spring and summer. Download FIG S5, TIF file, 1.1 MB. Copyright © 2021 Deel et al. 2021 Deel et al. https://creativecommons.org/licenses/by/4.0/ This content is distributed under the terms of the Creative Commons Attribution 4.0 International license.

10.1128/mSphere.00455-21.6 FIG S6 (A) Principal-coordinate analysis of the 16S rRNA spring and summer placement soil control communities using the Aitchison distance metric. Features driving clustering differences between seasons are labeled; PERMANOVA between seasons *P* = 0.001, pseudo-F (effect size) = 68.72, df = 1 with 999 permutations. (B) Taxa plots of the 16S rRNA data of soil controls collected within the first 21 days of decomposition in the spring and summer placements. Rare taxa include those with a mean relative abundance of 0.008 or lower. Download FIG S6, TIF file, 2.8 MB. Copyright © 2021 Deel et al. 2021 Deel et al. https://creativecommons.org/licenses/by/4.0/ This content is distributed under the terms of the Creative Commons Attribution 4.0 International license.

10.1128/mSphere.00455-21.7 FIG S7 (A and B) Accumulated precipitation (A) and humidity (B) data between day of placement and last day of collection for the spring and summer placements. Accumulated precipitation was calculated by adding average inches of precipitation per day, while accumulated humidity was calculated by adding average percent humidity per day. Weather data were collected from the Easterwood Airport Station using Weather Underground. FIG S7, TIF file, 1.5 MB Copyright © 2021 Deel et al. 2021 Deel et al. https://creativecommons.org/licenses/by/4.0/ This content is distributed under the terms of the Creative Commons Attribution 4.0 International license.

### Can we use microbial invasion in bone to estimate PMI?

Bacterial community composition became increasingly different from the initial rib bone community as decomposition progressed (Fig. 4A), with rate of change of community composition decreasing over ADD (Fig. 4B), indicating a repeatable succession of invading microbes. Furthermore, effect size calculations showed that ADD had the highest effect on beta diversity in nearly every case (particularly for the 16S rRNA data; Table S3). Therefore, these data may be useful for predicting PMI of remains in an advanced stage of decomposition, in which the ribs have at least skeletonized. Microbial eukaryotic community composition also changed during decomposition, but with a less distinguishable pattern compared to 16S rRNA data (Fig. S4). Because we detected microbial community differences for cadavers placed in different seasons (Fig. 4A), we tested whether season-specific PMI models performed better (i.e., produced a lower mean absolute error).

Random forest models using only the 16S rRNA ASV-level data from summer placement cadavers provided the most accurate models (i.e., lowest range of mean absolute errors [MAEs]). The most accurate model had MAEs ranging from 724 to 853 ADD over a total of 5,201 ADD for the summer data, which roughly equates to an error of ±39 days. Models including both the spring and summer placement data (i.e., the “combined” models) gave a higher range of MAEs, while models with just the spring placement data provided the highest range of MAEs within the 16S rRNA data set (Table 1). In the 18S rRNA data set, this pattern was the same. The lower mean absolute error (i.e., increased accuracy) of the summer placement over the spring placement in both 16S rRNA and 18S rRNA data sets may be explained by the wider sample collection time frame (Table S1). Perhaps the increased information gained from an extra 2 to 3 months of sampling for decedents placed in the summer allowed for the model to account for more variability. Despite relatively similar ADDs within each season, it is possible that once skeletonization occurs, a longer time frame of collection is more important than combined temperature and time (i.e., ADD). Another factor explaining this difference could be the placement of all spring subjects on the same day, whereas the placement of summer subjects ranged over approximately 2 weeks (Table S1). This unintended occurrence was due to the limited availability of decedents in the summer placement. This may have allowed the summer models to capture more variability than the spring models, giving more accurate predictions.

**TABLE 1:** Random forest regression modeling of amplicon data using features collapsed at different taxonomic levels^*a*^

Amplicon	Season(s)	Most accurate level	Range of MAEs	Top five important features	Range of importance
16S rRNA	Spring and summer (“combined”)	ASV	793.33–851.41	*Phyllobacteriaceae*, *Defluvibacter*, *Corynebacterium*, *Shinella*, *Devosia*	0.040–0.023
16S rRNA	Spring	ASV	872.02–1,074.76	*Gallicola*, *Cellulosimicrobium*, *Brachybacterium*, *Comamonas*, *Leucobacter*	0.075–0.042
16S rRNA	Summer	ASV	723.98–853.38	*Phyllobacteriaceae*, *Sphingopyxis*, *Alcaligenaceae*, *Devosia*, *Pseudaminobacter*	0.014–0.010
18S rRNA	Spring and summer (“combined”)	8	941.22–1,128.13	Eurotiomycetes, Sordariomycetes, Metazoa, Saccharomycetes, Tremellomycetes	0.067–0.033
18S rRNA	Spring	5	1,025.53–1,443.86	Mucoromycota, Metazoa, Vannellida, Eumetazoa, Dikarya	0.102–0.047
18S rRNA	Summer	7	820.67–1,083.95	Nematoda, Saccharomycotina, BOLA868, Alveolata, Eumetazoa	0.071–0.037

a Model accuracy is assessed using mean absolute error (MAE). The range of MAEs resulting from modeling at all taxonomic levels is reported. The top five most important features within each model are arranged from the most to least important, as determined by the random forest regression. Note that some important features were not able to be classified all the way down to the same taxonomic level at which the model was performed (e.g., Metazoa). Underlined features include those commonly important between model types. Note that there are no commonly important features in the 18S rRNA models due differences in the most accurate levels, whereas in the 16S rRNA models, all of the most accurate models were at the ASV level. MAEs for all levels are reported in Table S2.

10.1128/mSphere.00455-21.9 TABLE S2 Random forest regression modeling of 16S rRNA and 18S rRNA data using features collapsed at different taxonomic levels. Model accuracy is assessed using mean absolute error (MAE). The model with the lowest error within each season (spring and summer together, spring only, summer only) is in bold. Download Table S2, DOCX file, 0.02 MB. Copyright © 2021 Deel et al. 2021 Deel et al. https://creativecommons.org/licenses/by/4.0/ This content is distributed under the terms of the Creative Commons Attribution 4.0 International license.

Modeling results indicate that the 16S rRNA data are more accurate in estimating PMI than the 18S rRNA data (Table 1). Nearly all ranges of MAEs obtained using 18S rRNA data were higher than those obtained using 16S rRNA data when comparing the same combination of seasons (e.g., the range of combined 18S rRNA MAEs was higher than the range of combined 16S rRNA MAEs). Modeling results for both data sets at each taxonomic level are provided in Tables S2 and S3. At the ASV level, there are over three times as many features in the 16S rRNA data than the 18S rRNA data (5,708 versus 1,696, respectively). The 16S rRNA model may have been able to effectively use an increased number of features to produce a lower MAE. This may also be explained by a less defined trend of dissimilarity with increasing ADD in the 18S rRNA data (Fig. S4), indicating that the microbial eukaryotic community within decomposing bone is highly variable and less able to predict PMI. Further evidence of this is observed in the most accurate levels for 16S rRNA and 18S rRNA modeling (Table 1). Since the ASV level is the most accurate for all 16S rRNA models, this indicates that microbial succession within these data is defined well enough that the model is able to find patterns within these particular ASVs to predict PMI with some accuracy. This is also supported by effect size calculations in which host appears to have the highest effect on 18S rRNA beta diversity in four cases (Table S3).

Across models using 16S rRNA ASV data, there were two commonalities in the top five important features, *Phyllobacteriaceae* and *Devosia* (shown underlined in Table 1). These taxa are both within the order *Hyphomicrobiales* (also known as *Rhizobiales*) and were shown to increase in prevalence at higher ADDs, which likely contributes to their high importance in these models. For example, for summer placed cadavers between ADDs 592 and 2,414, *Phyllobacteriaceae* is only present in 3/11 samples with a total of 45 reads, while *Devosia* is prevalent in only 2/11 samples with a total of 100 reads. As decomposition progresses between ADDs 2,804 and 5,201, *Phyllobacteriaceae* and *Devosia* are present in all 12 samples with a total of 3,303 and 2,677 reads, respectively. This apparent trend across time suggests that these taxa may provide some useful information about the ecology of decomposed bone over time. For example, *Phyllobacteriaceae* consists of environmental (soil, water) and plant-associated bacteria that use oxygen as the terminal electron acceptor in respiratory metabolism (33). Perhaps increased porosity in the bone over decomposition contributes to higher levels of oxygen, allowing for this family to increase in prevalence. *Devosia*, a genus known for dominance in soil habitats, is known for encoding a large diversity of transporters that allow it to take up short peptides for satisfying nutritional needs (34). This may facilitate its use of the variety of nutrients that are provided in the dynamic decomposition environment more efficiently that other bacteria, allowing them to predictably thrive with increased decomposition. The ability to fix nitrogen may also play a role in the importance of these organisms. *Devosia* is a nitrogen-fixing bacterium (35), and *Phyllobacteriaceae* is closely related to organisms known for nitrogen fixation (36). Perhaps they are using collagen of the bone as a source of nitrogen (37) and increasing in prevalence as more collagen becomes exposed with higher levels of porosity.

In the 18S rRNA models, several important features were representative at a range of taxonomic levels that consist of broadly defined taxa, including fungi, metazoans such as nematodes, and amoebae, which can flourish in a wide variety of habitats (Table 1). Just in the combined model, features include yeasts and fungi that have been isolated from environments including humans (38), soil and freshwater (39), plant material (18), or a combination including several of these listed environments (40). Although this wide range of important features in the 18S rRNA models provides less defined information about the ecology of decomposed bone over time, it nevertheless provides a picture of the suite of microbial eukaryotes that inhabit decomposed bone.

### Conclusions and limitations.

This research demonstrates the potential use of postmortem bone microbial communities to predict time since death in human remains with PMIs of 9 months or less.

In the 16S rRNA spring and summer placement model, the lowest MAE of 793.33 roughly approximates to an error of ±34 days. Although much additional research is needed, this model has the potential to generate probative PMI estimates, and it certainly represents progress toward improving medicolegal death investigations. To put this into the context of investigations involving skeletal remains that have been decomposing within a similar time frame of this study (∼1 to 9 months), without other evidence, anthropologists are typically able to give PMI estimations with errors of several months or even years (41). Anthropologists typically provide PMI estimates in relative time based on qualitative information gathered from the death scene and the body itself. Otherwise, very few methods exist for estimating PMI within this time frame.

While these data represent an initial attempt to characterize the succession of postmortem bone microbial communities using a controlled research design at a decomposition research facility, there remain limitations worth addressing. First, the sample size (48 rib bones from 6 human individuals) is small. Large numbers of willfully donated human decedents are difficult to obtain for the purposes of decomposition research despite the existence of decomposition research facilities, and not all decedents are available for destructive sampling, which is required for skeletal DNA analysis. This lack of biological replicates often pushes researchers toward the use of animal proxies, which do not often decompose in a similar manner to human remains (42). We opted to use human decedents to make this research more applicable to forensic contexts involving human remains. Although we did not see host having a larger effect than ADD or season in nearly every case (Table S3), we recognize that an increase in decedent sample size would mitigate any nondetectable host-host variations. The limited availability of human decedents also meant that some were frozen before placement. While there is some evidence that freeze-thawing affects the decay of soft tissues in rats (43), there is no known evidence that this affects the long-term microbial decomposition of human bone. Future studies should focus on a more consistent protocol (i.e., no individuals should ever be frozen). Second, there were some minor differences between the spring and summer seasons that contributed to our limitations. There was discordance in the placement protocol between spring and summer seasons such that in the spring, all decedents were placed on the same day, while in the summer, decedents were placed on different days. This may have resulted in a reduction in power for the spring season models by effectively making each placed decedent a pseudoreplicate. Furthermore, the spring placement cohort was uncaged, whereas the summer cohort was caged to protect from scavengers. Lastly, the research design implemented here makes the assumption that all ribs have similar microbial communities at each time point. Two ribs collected from the same donor on the same time point indicate that this may not be true, and others have shown bone microbial community differences related to spatial positioning and bone type (13). Regardless of the validity of this assumption, random forest models were able to overcome differences in microbial community composition related to rib positioning.

To overcome these limitations, future research will attempt to increase sample sizes, stagger the placement of decedents, use only never-frozen individuals, and better characterize microbial differences by bone location/type. Moreover, to better understand the ecological significance of predictive taxa and elucidate their potential role in skeletal degradation, future research will include other types of measured edaphic (e.g., soil moisture, phosphate, nitrate, and microbial biomass) and skeletal parameters (e.g., organic composition, histological indicators of microbial damage, and other indicators of skeletal degradation).

Despite these limitations and the observed variation in diversity, taxonomic composition, and important taxa between seasons and within PMI models, the model using data from both placements can still estimate PMI at an accuracy that is better than the currently used methods for skeletonized remains. As noted in reference 15 and supported by findings in this study, seasonality is likely important for developing a robust microbial clock to estimate PMI. This key point can now be extended to studies using decomposed bone. With future studies, the microbial ecology of decomposed bone and the surrounding environment can be further elucidated, providing insight into this unique ecosystem as well as new potential means for more accurately estimating PMI.

## MATERIALS AND METHODS

### Decedent placement and sampling.

Research was conducted in collaboration with the Southeast Texas Applied Forensic Science Facility (STAFS), previously known as the Applied Anatomical Research Center, an anthropological research center in Huntsville, TX. Willfully donated human decedents were placed outdoors, unclothed, and in the supine position to decompose under natural conditions. Three decedents were placed on 14 April 2016, which are called our spring placement bodies.

For our summer placement, decedents were placed outdoors as they became available to reduce time in cooled storage, during which time the decomposition process is slowed but not completely halted (44). As a result, two summer bodies were placed on 25 August 2016, while the third was placed on 16 September 2016. While some decedents were frozen, there is no known evidence that this affects the long-term microbial decomposition of human bone. Due to discordance between seasonal placements within the facility, the spring cohort was uncaged, whereas the summer cohort was caged and protected from scavengers. Sample collection was conducted in a similar manner as in Damann et al. (7). Collection of rib bones began once decomposition progressed sufficiently such that little dissection was needed. There was not a requirement for the rib to be fully exposed before collection. We were unable to calculate the percentage of rib exposure due to a lack of photos during sample collection, as per policy of the anthropological facility. It is important to note that sample collection of the spring placement decedents ranged from 16 May 2016 to 11 October 2016, and sample collection of the summer placement decedents ranged from 22 September 2016 to 8 June 2017. Therefore, while “spring” and “summer” both indicate certain times of the year, in this case they only refer to when the decedents were placed and not when samples were collected within other seasons of the year. Right and left lower ribs were selected by the field sampler based on ease of collection (i.e., ribs were collected based on the level of dissection required, with preference toward those requiring the least dissection). Samples were collected approximately every 3 weeks for a total of 8 bones from each body (48 overall), with one exception, in which two ribs were mistakenly collected from the same decedent, resulting in one subject with nine time points and another subject with only seven time points. Each rib was individually bagged and immediately frozen at −10°C and then stored until shipping to Colorado State University for processing. Accumulated degree day (ADD) was estimated using weather data provided by the National Centers for Environmental Information (https://www.ncdc.noaa.gov/). Degree day on the day of placement was not included, and a base temperature of 0°C was used. ADD was calculated by adding together all average daily temperatures above 0°C for all prior days of decomposition, as in Megyesi et al. (10). A sampling summary is provided in Table S1.

### Rib bone processing.

The rib bones were shipped on dry ice to Colorado State University and then stored at −20°C until processing. Spring collections were processed in December 2016, and summer collections were processed in August 2017. A fume hood was cleaned with 20% bleach solution before processing and between each bone sample. Each rib was mechanically abraded with a handheld Dremel drill to remove any tissue and superficial layers of cortical bone. An approximate 40 mm by 15 mm section of bone was removed from the rib angle. The remainder of the samples were stored at −20°C.

To remove microbial DNA from the exterior of ribs, each sample was weighed and UV irradiated at 254 nm for 30 min on each side. Each sample was wiped down with 3% bleach and then abraded again with the Dremel drill to ensure removal of the outer layer of bone. The sample was then divided into three equal segments, each of which were weighed and placed into a tube. Two segments were stored at −20°C for potential future use, while the remaining sample was pulverized in a sterile Waring MC2 blender cup. The cup was washed and soaked in bleach for 3 min between each sample. Each of the bone powders was placed into a clean tube for extraction.

### Extraction and purification.

DNA was extracted from 0.2 to 0.5 g of pulverized bone. The samples were demineralized and lysed using 30 μl of 10% sodium dodecyl sulfate (SDS), 20 μl proteinase K, and 500 μl 0.5 M ethylenediaminetetraacetic acid (EDTA) (45). The samples were vortexed for 2 s and placed on a heating block at 55°C for 1 h, with additional 2-s vortexes every 15 min. The lysed samples were centrifuged at 10,000 × *g* for 1 min at room temperature. The supernatant was removed, measured, and placed into a clean tube. The pellets were kept and frozen at −20°C. Fifteen extraction blanks were included to identify any potential contamination.

DNA was purified using the PowerSoil DNA isolation kit from MoBio (Carlsbad, CA) with a modified protocol. Solution C4 was added to the lysed supernatant at twice the volume of the supernatant. The addition of solution C5 and centrifugation was performed one extra time. Occasionally, additional centrifugation was used to pass the remaining supernatant through the filter when it became clogged with bone debris. All other steps were performed as per the manufacturer’s instructions.

### Amplification and sequencing.

The bacterial communities of the samples and extraction negative controls were characterized using the 16S ribosomal RNA (rRNA) V4 and 18S rRNA gene regions. Standard primer pairs and protocols according to the Earth Microbiome Project were followed (46). Sequencing was performed on the MiSeq platform for the 16S rRNA gene region (2 × 150-bp reads) and the HiSeq platform for the 18S rRNA gene region (2 × 150-bp reads) using standard protocols (Illumina, San Diego, CA, USA) at the University of California San Diego IGM Genomics Center.

### Data analysis.

Sequencing information was uploaded onto QIITA (study 11553), an open-source microbial study management platform (47). Due to poor reverse read quality, only the raw forward read sequencing files were downloaded and imported into QIIME2 software 2018.4 for analysis (48).

### 16S rRNA preprocessing.

Reads were demultiplexed using uniquely assigned barcodes. Sequences were quality filtered using Deblur with a trim length of 150 bp. Taxonomy was assigned using the naive Bayes classifier, which was trained on Greengenes 13_8 99% operational taxonomic units (OTUs) (49). After removal of mitochondria and chloroplasts, a tree was generated by inserting fragment sequences using SEPP into the Greengenes 13_8 reference phylogeny using the QIIME2 plugin. For core metric phylogenetic analyses (50), the data were rarefied to 17,098 reads (see “Quality of Amplicon Sequence Data,” above), which removed all 15 negative extraction controls and 3 samples with low numbers of reads (007.R11, 011.L09, and 064.L12). These samples were removed from all analyses.

### 18S rRNA preprocessing.

Similar to 16S rRNA processing, reads were demultiplexed using uniquely assigned barcodes. Sequences were quality filtered using Deblur with a trim length of 150 bp. Reference sequences were obtained from the SILVA database at https://www.arb-silva.de/download/archive/qiime (SILVA_132_QIIME_release/rep_set/rep_set_18S_only/99/silva_132_99_18S.fna), which was then imported as a QIIME2 artifact. Taxonomy was assigned using a classifier trained on the full-length SILVA 132 99% 18S database (51) using the feature-classifier QIIME2 plugin (52). Sequence data representing nonmicrobial taxa were filtered out, including Archaeplastida, Arthropoda, Chordata, Mollusca, *Bacteria*, and unassigned taxa. While there were many unclassified eukaryotes (50 in total), BLAST (53) results of these features generally consisted of yeasts, other fungi, and nematodes (i.e., there were no obvious nonmicrobial taxa). A phylogenetic tree using FastTree (54) and MAFFT alignment was generated using the phylogeny plugin for core metric analysis. For core metric phylogenetic analyses (50), the data were rarefied to 214,940 reads, resulting in a loss of 10 samples (067.R12, 067.L11.march, 065.L11, 065.L09, 065.L10, 064.R09, 024.L10, 007.R10, 007.L09, 011.L09).

### Diversity estimates.

A linear mixed effects model was used to consider alpha and beta diversity over time (fixed effect), and a random effect for subject was included to account for repeated measurements on the same subject across time. Alpha diversity was assessed using the rarefied data with Faith’s phylogenetic diversity metric. The linear mixed effects, plot-feature-volatility, and volatility visualizers in the longitudinal plugin in QIIME2 were used to identify significant differences in community richness across ADD and between seasons. In addition to the linear mixed effects model, beta diversity was assessed using weighted and unweighted UniFrac distances (55), which were visualized using principal-coordinate analysis (PCoA) and the data visualization tool EMPeror (56, 57). Effect sizes between groups (season, hosts, and the first and last ADDs) were calculated using kruskal_effsize() in the rstatix() package (58) for alpha diversity, and the pseudo-F output from the permutational multivariate analysis of variance (PERMANOVA) (59) test in the beta-group-significance visualizer in the diversity plugin in QIIME2 was used for beta diversity.

### Identifying core features and features different between seasons.

Initial exploration of features within and between seasons was performed using taxa plots. Relative abundance taxa bar plots were generated using the taxonomically filtered and rarefied data (see below for software and packages used). To aid in interpretation of the taxa plots, taxa present at low relative abundances were lumped into rare taxa. These taxa were identified using mean relative abundances that ranged from 0.002 to 0.008 across the entire data set (see each plot for the specific number). Core taxa within each season were identified using the core-feature visualizer in the feature-table plugin in QIIME2 with the default setting of 0.5 as the minimum fraction of samples that a feature must be observed in to be considered a core feature. Taxa between seasons were compared using the ANCOM (60) visualizer in the composition plugin in QIIME2. Significance between beta diversity of seasons was determined using a nonpairwise PERMANOVA test with the beta-group-significance visualizer in QIIME2.

### Source tracking.

To predict the environmental source of internal rib bacteria, initial analysis in QIIME2 (60) as well as the SourceTracker2 package (61) were used. Although no skin and soil samples were collected for this study, the same subjects from both spring and summer placements were included in another study in which skin and soil samples were collected daily for the first 21 days of decomposition. Amplification and sequencing of the 16S rRNA gene were performed following protocols in the Earth Microbiome Project (61). Fresh sources, those representing the unique, nondecomposer microbiome of the subject, include decedent skin (of the hip and face) and decedent soil (near the hip and face) samples collected on the day of placement and the day after placement (days 1 and 2, respectively), as well as all soil control (non-decedent-associated) samples. Note that previous studies have found that the human microbiome is stable for up to 2 days after death (62). Samples of the advanced decay community similarly include skin and hip samples of the decedent skin and decedent soil collected on days 19, 20, and 21. Reference hit biom tables (trimmed at 150 bp) that included the samples to be used for source tracking were downloaded from QIITA study 11271. These biom files were imported as QIIME2 artifacts using BIOMV210Format and merged into a single feature table with the rib samples. Similarly, the reference-hit.seqs.fa files in QIITA study 11271 corresponding to source tracking samples were downloaded and imported as QIIME2 artifacts and merged into a single representative sequences file with the rib samples. Taxonomy was assigned similarly to the bone samples, using the naive Bayes classifier, which was trained on Greengenes 13_8 99% OTUs (62). After filtering out mitochondria and chloroplasts, a fragment-insertion SEPP tree was generated for use in core metric phylogenetic analyses. Diversity analyses of the rib and source communities were performed using the core metrics phylogenetic pipeline in the diversity plugin in QIIME2 at a sequencing depth of 4,548. This was to validate that the skin and soil sources were different from each other and that the fresh and advanced decay communities were distinct. Alpha and beta diversity were assessed using the Shannon and UniFrac metrics, respectively. Significances between alpha diversity of sources and rib communities were calculated using the Wilcox test in the stat_compare_means() function in R (63), and significance between beta diversity of sources and the rib communities was determined using pairwise PERMANOVA in the beta-group-significance visualizer in QIIME2. To use SourceTracker2 (61), the rarefied feature table was exported as a BIOM 2.1.0 table, and source predictions were generated using the Gibbs function. For analysis of the soil control data only (see Fig. S6), relative abundance taxa plots were generated using a feature table rarefied at 4,548 reads per sample. The DEICODE plugin (63) in QIIME2 and the Aitchison distance metric were used to generate a biplot with features that influence the principal-component axes to help identify taxa that were driving differences between seasonal placements.

Note that SourceTracker2 analysis was not performed using the 18S rRNA data; this is due to exploration of diversity and modeling results indicating that the 16S rRNA communities were less noisy and more predictive of PMI, which directed the source tracking investigation to focus only on the 16S rRNA data.

Final result plots were generated using the packages phyloseq (64), qiime2R (65), Tidyverse (66), R ColorBrewer (67), randomcoloR (68), and ggpubr (69) in R software 3.5.1 (63).

### Model testing.

Feature abundance data were used to generate postmortem interval (PMI) prediction models using random forest regressors. The same rarefied feature tables that were produced during diversity analyses were converted to BIOM 2.1.0 tables and used for modeling. K-fold cross validation (nonnested) was performed so that the data were separated by individual, and data from the same individual were used in either the training (model-fitting) or the testing (postmortem interval-predicting) set, but not both. The number of estimators used in each model was 1,000, and hyperparameter tuning was used to refine the model. All bootstrapping was set to false in the hyperparameter tuning grid. Mean absolute error, the average deviation between predicted and observed values, was used to measure the accuracy of the model. These methods were applied using data from both seasonal placements (spring and summer, termed “combined”), as well as only spring or only summer to determine if separate models could more accurately predict PMI. For each model type, models were produced at each taxonomic level to determine which was the most predictive. This was done by collapsing the rarefied feature table at all taxonomic levels in QIIME2 and then performing the same modeling methods as described above for each level. Since random forest innately assigns importance to features used in modeling, these data were extracted from the models and used to determine which features were most important in predicting PMI. Modeling and the extraction of important features was done with the Python machine learning package scikit-learn 19.0 (70).

### Data availability.

All data are available in QIITA study 11553, and all analysis codes are provided at https://github.com/Metcalf-Lab/bone_2020_Deel.
