# Supplementary material for: A Pilot Study of Microbial Succession in Human Rib Skeletal Remains during Terrestrial Decomposition
Source: mSphere. 2021 Jul 14;6(4):e00455-21. doi: 10.1128/mSphere.00455-21 (PMC8386422; doi:10.1128/mSphere.00455-21)
Supplement: TABLE S1 [file msphere.00455-21-st001.docx]

**Table S1.** Summary of rib bones collected from human cadavers placed at STAFS. The beginning date of the range of collection indicates the first known occurrence of rib exposure.

| Season | Body ID | Day of placement | Advanced decay reached | Date range of collection | Ribs collected | ADD range |
| --- | --- | --- | --- | --- | --- | --- |
| Spring | 007 | 4/15/16 | 4/25/16 | 5/16/16 – 10/11/16 | L9-12, R9-12 | 708-4821 |
|  | 011 | 4/15/16 | 4/25/16 | 5/16/16 – 10/11/16 | L8-12, R8-10, R12 | 708-4821 |
|  | 024 | 4/15/16 | 4/25/16 | 5/16/16 – 10/11/16 | L9-12, R9, R11-12 | 708-4821 |
| Summer | 064 | 8/25/16 | 9/02/16 | 9/22/16 – 5/05/17 | L9-12, R9-12 | 592-4756 |
|  | 065 | 8/25/16 | 9/02/16 | 10/4/16 – 5/05/17 | L9-12, R8-11 | 877-4756 |
|  | 067 | 9/16/16 | 9/23/16 | 11/01/16 – 6/08/17 | L9-12, R9-12 | 1151-5201 |
