# Supplementary figures and images for: A Pilot Study of Microbial Succession in Human Rib Skeletal Remains during Terrestrial Decomposition
Source: mSphere. 2021 Jul 14;6(4):e00455-21. doi: 10.1128/mSphere.00455-21 (PMC8386422; doi:10.1128/mSphere.00455-21)

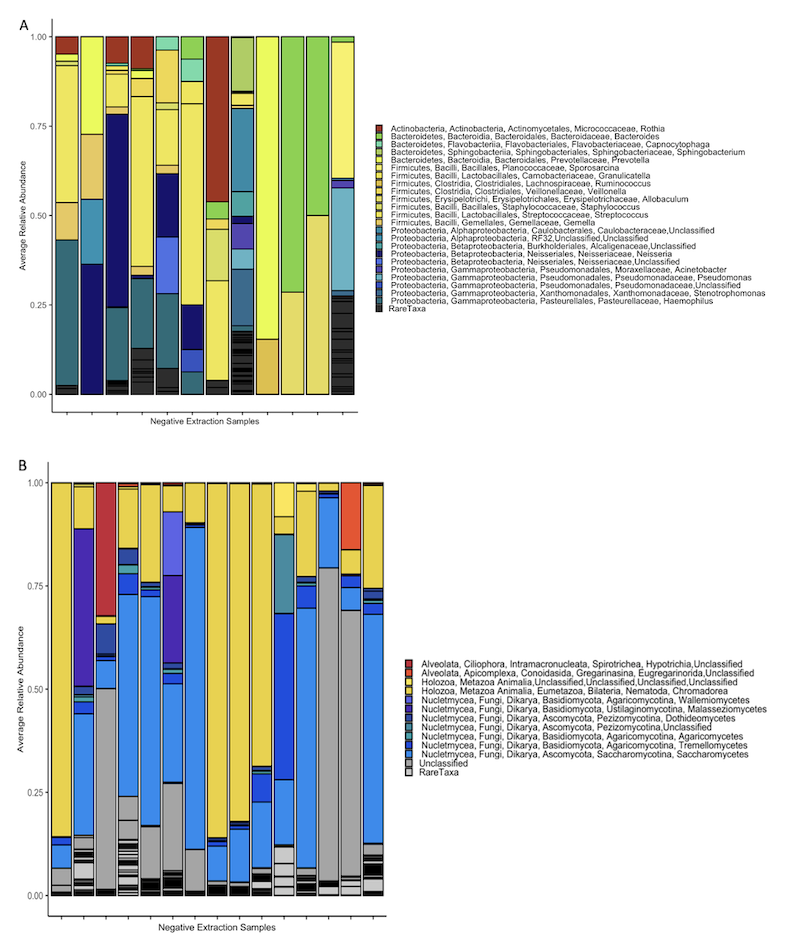

Supplement: FIG S1 [file msphere.00455-21-sf001.tif]

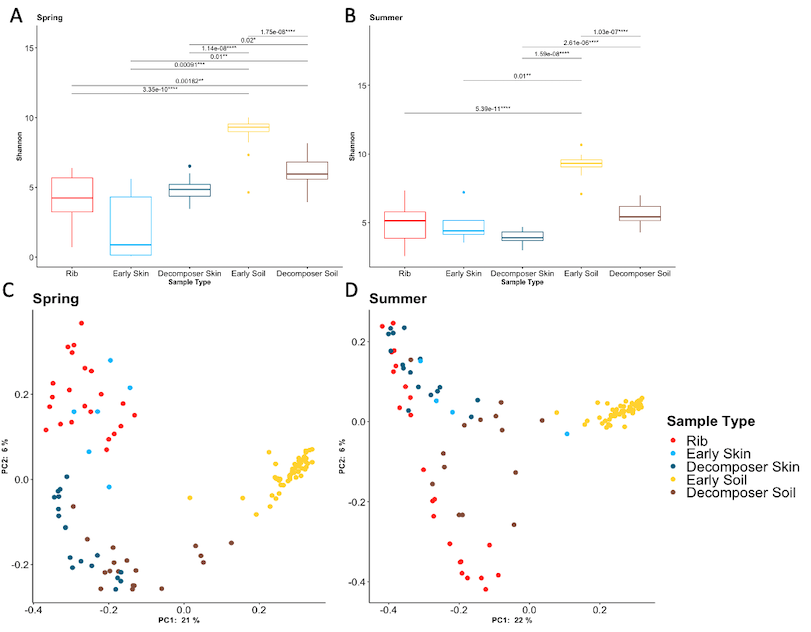

Supplement: FIG S2 [file msphere.00455-21-sf002.tif]

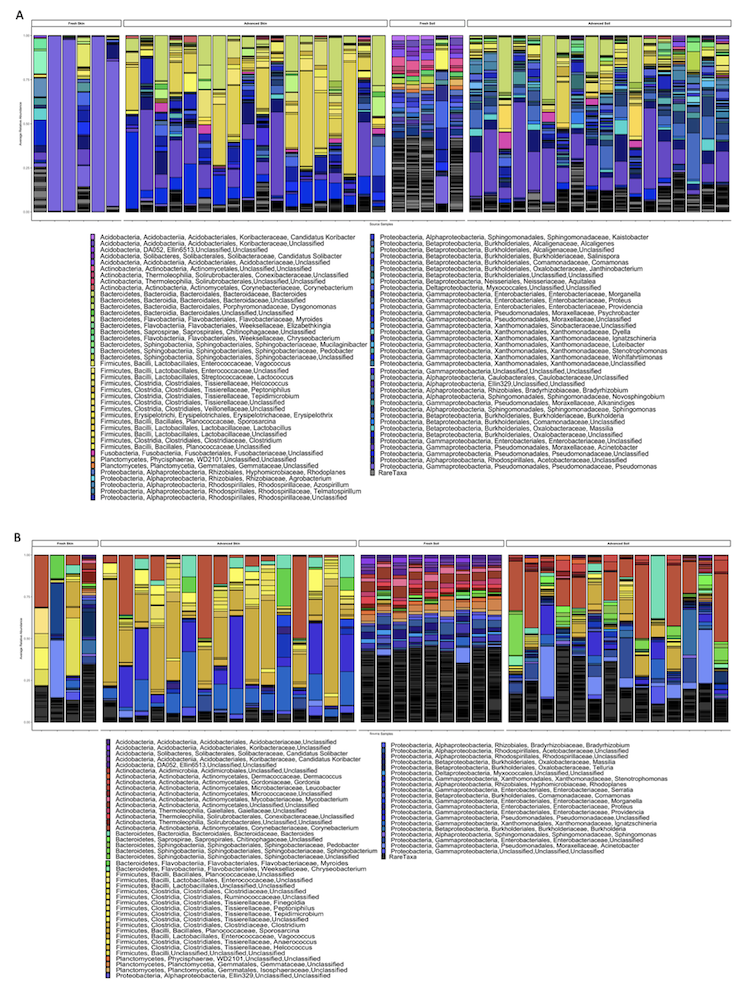

Supplement: FIG S3 [file msphere.00455-21-sf003.tif]

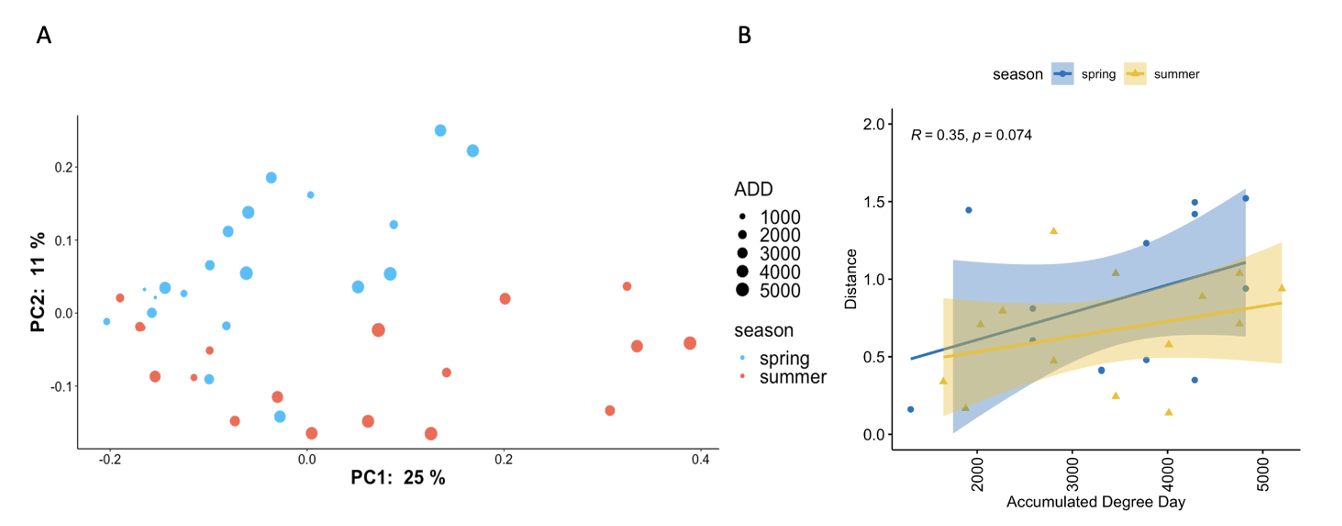

Supplement: FIG S4 [file msphere.00455-21-sf004.tif]

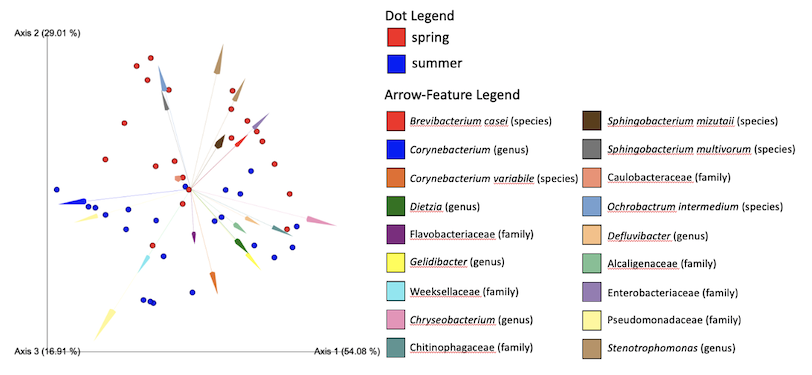

Supplement: FIG S5 [file msphere.00455-21-sf005.tif]

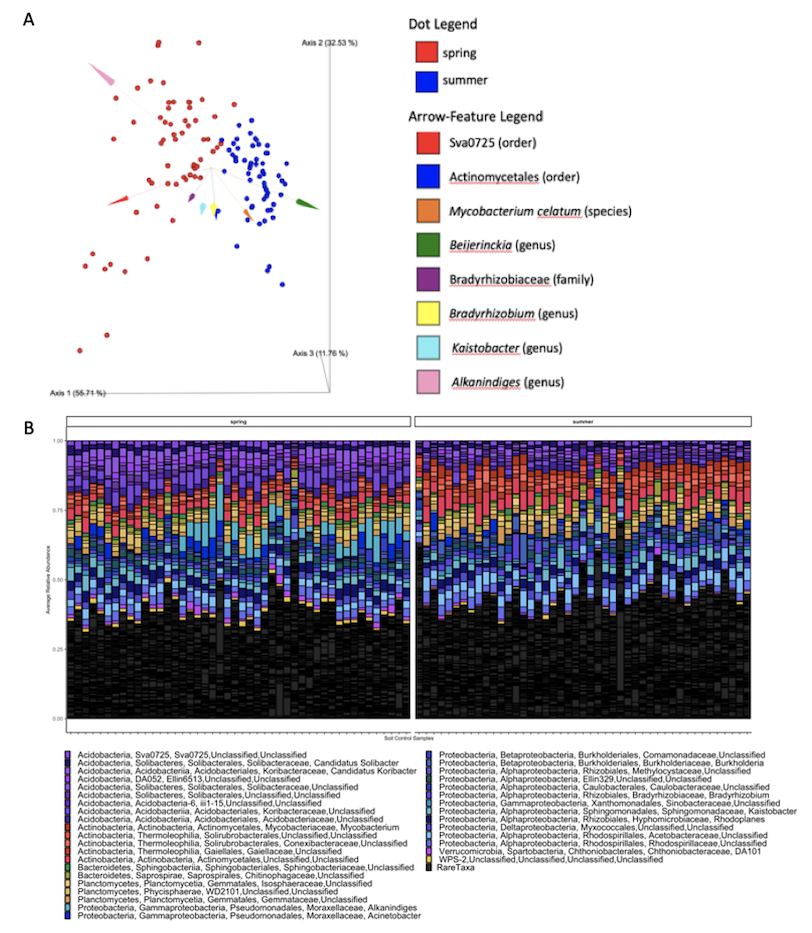

Supplement: FIG S6 [file msphere.00455-21-sf006.tif]

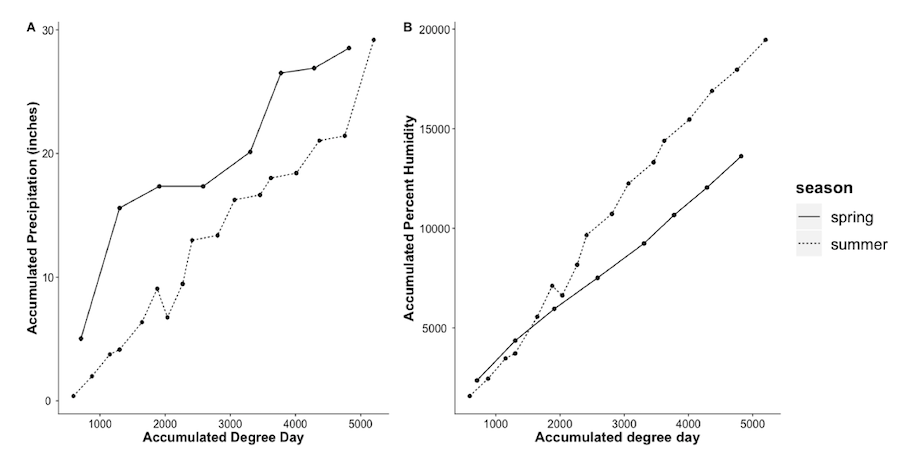

Supplement: FIG S7 [file msphere.00455-21-sf007.tif]
