# Supplementary material for: A Pilot Study of Microbial Succession in Human Rib Skeletal Remains during Terrestrial Decomposition
Source: mSphere. 2021 Jul 14;6(4):e00455-21. doi: 10.1128/mSphere.00455-21 (PMC8386422; doi:10.1128/mSphere.00455-21)
Supplement: TABLE S3 [file msphere.00455-21-st003.docx]

| **Data Type** | **Season(s)** | **Diversity Metric** | **Grouped by** | **Effect Size** |
| --- | --- | --- | --- | --- |
| 16S rRNA | Spring and Summer | Faith’s PD | Season | 0.02 |
|  |  |  | Host | 0.12 |
|  |  |  | 1^st^ and last ADD | 0.31 |
|  |  | Shannon | Season | 0.05 |
|  |  |  | Host | 0.08 |
|  |  |  | 1^st^ and last ADD | 0.44 |
|  |  | Unweighted UniFrac | Season | 2.32 |
|  |  |  | Host | 1.73 |
|  |  |  | 1^st^ and last ADD | 4.22 |
|  |  | Weighted UniFrac | Season | 2.42 |
|  |  |  | Host | 1.78 |
|  |  |  | 1^st^ and last ADD | 5.20 |
|  | Spring | Faith’s PD | Host | 0.24 |
|  |  |  | 1^st^ and last ADD | 0.13 |
|  |  | Shannon | Host | 0.07 |
|  |  |  | 1^st^ and last ADD | 0.37 |
|  |  | Unweighted UniFrac | Host | 1.46 |
|  |  |  | 1^st^ and last ADD | 2.12 |
|  |  | Weighted UniFrac | Host | 1.77 |
|  |  |  | 1^st^ and last ADD | 1.41 |
|  | Summer | Faith’s PD | Host | 0.10 |
|  |  |  | 1^st^ and last ADD | 0.79 |
|  |  | Shannon | Host | 0.15 |
|  |  |  | 1^st^ and last ADD | 0.50 |
|  |  | Unweighted UniFrac | Host | 1.66 |
|  |  |  | 1^st^ and last ADD | 3.45 |
|  |  | Weighted UniFrac | Host | 1.40 |
|  |  |  | 1^st^ and last ADD | 6.24 |
| 18S rRNA | Spring and Summer | Faith’s PD | Season | 0.04 |
|  |  |  | Host | 0.22 |
|  |  |  | 1^st^ and last ADD | 0.73 |
|  |  | Shannon | Season | 0.04 |
|  |  |  | Host | 0.09 |
|  |  |  | 1^st^ and last ADD | 0.73 |
|  |  | Unweighted UniFrac | Season | 2.90 |
|  |  |  | Host | 2.44 |
|  |  |  | 1^st^ and last ADD | 2.53 |
|  |  | Weighted UniFrac | Season | 0.85 |
|  |  |  | Host | 2.32 |
|  |  |  | 1^st^ and last ADD | 0.60 |
|  | Spring | Faith’s PD | Host | 0.07 |
|  |  |  | 1^st^ and last ADD | 0.80 |
|  |  | Shannon | Host | 0 |
|  |  |  | 1^st^ and last ADD | 0.80 |
|  |  | Unweighted UniFrac | Host | 1.56 |
|  |  |  | 1^st^ and last ADD | 1.67 |
|  |  | Weighted UniFrac | Host | 0.80 |
|  |  |  | 1^st^ and last ADD | 0.20 |
|  | Summer | Faith’s PD | Host | 0.41 |
|  |  |  | 1^st^ and last ADD | 0.60 |
|  |  | Shannon | Host | 0.33 |
|  |  |  | 1^st^ and last ADD | 0.60 |
|  |  | Unweighted UniFrac | Host | 2.38 |
|  |  |  | 1^st^ and last ADD | 1.28 |
|  |  | Weighted UniFrac | Host | 6.10 |
|  |  |  | 1^st^ and last ADD | 4.24 |
