# Supplementary material for: A Pilot Study of Microbial Succession in Human Rib Skeletal Remains during Terrestrial Decomposition
Source: mSphere. 2021 Jul 14;6(4):e00455-21. doi: 10.1128/mSphere.00455-21 (PMC8386422; doi:10.1128/mSphere.00455-21)
Supplement: TABLE S2 [file msphere.00455-21-st002.docx]

**Table S2.** Random forests regression modeling of 16S rRNA and 18S rRNA data using features collapsed at different taxonomic levels. Model accuracy is assessed using mean absolute error (MAE). The model with the lowest error within each season (spring and summer together, spring only, summer only) is in bold.

| **Data type** | **Season** | **Level** | **MAE** |
| --- | --- | --- | --- |
| 16S rRNA | Spring and summer | **ASV** | **793.33** |
|  |  | L7 | 822.28 |
|  |  | L6 | 822.37 |
|  |  | L5 | 851.41 |
|  |  | L4 | 839.88 |
|  |  | L3 | 842.20 |
|  |  | L2 | 807.77 |
|  | Spring | **ASV** | **872.02** |
|  |  | L7 | 904.18 |
|  |  | L6 | 884.32 |
|  |  | L5 | 941.63 |
|  |  | L4 | 1025.01 |
|  |  | L3 | 987.59 |
|  |  | L2 | 1074.76 |
|  | Summer | **ASV** | **723.98** |
|  |  | L7 | 729.09 |
|  |  | L6 | 746.90 |
|  |  | L5 | 788.10 |
|  |  | L4 | 834.82 |
|  |  | L3 | 853.38 |
|  |  | L2 | 778.17 |
| 18S rRNA | Spring and summer | ASV | 1013.78 |
|  |  | L12 | 952.92 |
|  |  | L11 | 963.61 |
|  |  | L10 | 964.84 |
|  |  | L9 | 966.57 |
|  |  | L8 | 956.64 |
|  |  | **L7** | **941.22** |
|  |  | L6 | 1095.19 |
|  |  | L5 | 1040.01 |
|  |  | L4 | 1125.68 |
|  |  | L3 | 1128.13 |
|  |  | L2 | 1042.47 |
|  | Spring | ASV | 1128.14 |
|  |  | L12 | 1044.26 |
|  |  | L11 | 1074.59 |
|  |  | L10 | 1084.54 |
|  |  | L9 | 1110.03 |
|  |  | L8 | 1113.28 |
|  |  | L7 | 1187.64 |
|  |  | L6 | 1202.75 |
|  |  | **L5** | **1025.53** |
|  |  | L4 | 1168.03 |
|  |  | L3 | 1181.39 |
|  |  | L2 | 1443.86 |
|  | Summer | ASV | 990.91 |
|  |  | L12 | 877.59 |
|  |  | L11 | 864.45 |
|  |  | L10 | 858.58 |
|  |  | L9 | 870.40 |
|  |  | L8 | 886.16 |
|  |  | **L7** | **820.67** |
|  |  | L6 | 867.09 |
|  |  | L5 | 942.52 |
|  |  | L4 | 904.18 |
|  |  | L3 | 958.75 |
|  |  | L2 | 1083.95 |
